# Clinicians' evaluations of, endorsements of, and intentions to use practice guidelines change over time: a retrospective analysis from an organized guideline program

**DOI:** 10.1186/1748-5908-4-34

**Published:** 2009-06-28

**Authors:** Melissa Brouwers, Steven Hanna, Mona Abdel-Motagally, Jennifer Yee

**Affiliations:** 1 Departments of Oncology and Clinical Epidemiology and Biostatistics, McMaster University and Program in Evidence-based Care, Cancer Care Ontario, Hamilton, Ontario, Canada; 2 Department of Clinical Epidemiology and Biostatistics, McMaster University, Hamilton, Ontario, Canada; 3 McMaster University, Hamilton, Ontario, Canada; 4 Sunnybrook Hospital, Toronto, Ontario, Canada

## Abstract

**Purpose:**

Clinical practice guidelines (CPGs) can improve clinical care but uptake and application are inconsistent. Objectives were: to examine temporal trends in clinicians' evaluations of, endorsements of, and intentions to use cancer CPGs developed by an established CPG program; and to evaluate how predictor variables (clinician characteristics, beliefs, and attitudes) are associated with these trends.

**Design and methods:**

Between 1999 and 2005, 756 clinicians evaluated 84 Cancer Care Ontario CPGs, yielding 4,091 surveys that targeted four CPG quality domains (rigour, applicability, acceptability, and comparative value), clinicians' endorsement levels, and clinicians' intentions to use CPGs in practice.

**Results:**

Time: In contrast to the applicability and intention to use in practice scores, there were small but statistically significant annual net gains in ratings for rigour, acceptability, comparative value, and CPG endorsement measures (p < 0.05 for all rating categories). Predictors: In 17 comparisons, ratings were significantly higher among clinicians having the most favourable beliefs and most positive attitudes and lowest for those having the least favourable beliefs and most negative attitudes (p < 0.05). Interactions Time × Predictors: Over time, differences in outcomes among clinicians decreased due to positive net gains in scores by clinicians whose beliefs and attitudes were least favorable.

**Conclusion:**

Individual differences among clinicians largely explain variances in outcomes measured. Continued engagement of clinicians least receptive to CPGs may be worthwhile because they are the ones showing most significant gains in CPG quality ratings, endorsement ratings, and intentions to use in practice ratings.

## Introduction

Evidence-based clinical practice guidelines (CPGs) are knowledge products defined as systematically developed statements aimed to assist clinicians and patients in making decisions about appropriate healthcare for specific clinical circumstances [1]. Health service researchers have debated the extent to which CPGs have been effective in influencing practice or clinical outcomes [2-4]. Systematic reviews by Grimshaw and colleagues suggest that CPGs, or similar statements, do on average influence both the processes and outcomes of care, although the effect sizes tend to be modest [5-7].

Intentions to use CPG recommendations and their ultimate adoption are complex processes that may depend on many factors in addition to the validity of the recommendations. For example, while faithfulness to evidence-based principles is important, other non-methodological factors believed to influence the uptake of CPGs include adopters' perceptions of the CPG characteristics and messages and the CPG development process, actual and perceived facilitators and barriers to implementation, and factors related to norms and the practice context [2,8-15]. For example, consistent with a social influence perspective, evidence has shown greater compliance with CPGs perceived to be compatible with existing norms and not demanding changes in existing practices [14].

In addition, however, Brouwers *et al*. found that variability in oncologists' endorsement of and intentions to use cancer CPGs could be attributed more to differences among clinicians and variations in their perceptions of the CPG product, rather than to differences in the CPGs themselves [9]. Indeed, attitudes and beliefs can be extremely powerful. Whereas attitudes are evaluations of an object (*e.g.*, like versus dislike), beliefs are the perceived associations between an attitude object and various attributes, which may or may not have evaluative implications [16,17]. Together, an individual's attitudes and beliefs can have a significant impact on how information is gathered, encoded, and attributed. Indeed, decades-long research in the social psychological fields of social cognition, attitudes, intentions, and behavior demonstrate that the process of deciding what information is relevant and how one interprets information are guided by preexistent expectations [16-18]. Further, beliefs often provide the cognitive support for attitudes which can directly influence intentions to act and can influence actions themselves [16-18].

Research has often considered issues of guideline quality, users' beliefs and attitudes both independently and at one time. This work has been extremely important in identifying factors that more or less affect how CPGs are perceived by intended users and in predicting their uptake. Further, research examining factors related to the CPG uptake by clinicians has traditionally explored CPGs in contexts separate from a formal healthcare system in which they operate. In contrast, our interests were to design the research paradigm that explored issues of guideline quality, beliefs, and attitudes in an established CPG enterprise that is integrated into a formal healthcare system, and to assess the extent to which various factors are influenced by time. Understanding this will provide greater direction regarding efforts to promote utilization of CPGs into practice and healthcare systems decisions. This is pertinent given there are many CPGs available, and that CPG recommendations can change quickly in response to the proliferation with which new evidence and care options emerge.

The specific study objectives were to: examine temporal trends in clinicians' evaluations of, endorsements of, and their intentions to use cancer CPGs developed by an established cancer CPG program; and evaluate how clinician characteristics and clinician beliefs and attitudes are associated with these trends.

## Methods

### Context

The Cancer Care Ontario Program in Evidence-based Care (PEBC) in Ontario, Canada, a provincial CPG cancer system initiative, served as the context for this study. The PEBC CPGs are used to facilitate practice, guide provincial and institutional policy, and enable access to treatments in the publicly funded provincial healthcare system [19-21]. The PEBC is one component of a larger formalized cancer system defined by data and monitoring of system performance, evidence-based knowledge and best practices, transfer and exchange of this knowledge, and strategies to leverage implementation of knowledge. The work of the PEBC targets primarily the knowledge and transfer components of this system.

The PEBC methods include the systematic review of clinical oncology research evidence by teams, i.e., disease site groups (DSGs) comprised of clinicians (medical oncologists, radiation oncologists, surgeons, and other medical specialists) and methodological experts; interpretation and consensus of the evidence by the team; development of recommendations; and formal standardized external review of all draft CPGs [19,20,22]. The external review process involves disseminating draft CPGs and a validated survey, Clinicians' Assessments of Practice Guidelines in Oncology (CAPGO), to a sample of clinicians for whom the CPG is relevant. To create an appropriate sample, defining features of the CPG (*e.g.*, topic, modality of care, disease site) are matched with professional characteristics of clinicians held in a comprehensive database of clinicians involved in cancer care in the province. The ultimate number of clinicians invited to review varies considerably; guidelines targeting less common cancers tend to be small (<25 clinicians for sarcoma topics) compared to guidelines targeting more common guidelines (>100 clinicians lung cancer topics). Reminders are sent to non-responders at two weeks (postcard) and four weeks (full package), with closure of the review process typically between weeks seven and eight. During this time period, the average return rate was 51%. The external review methodology has been discussed at length elsewhere [9,22-24].

In this study, a retrospective analysis was conducted on data gathered in the formal external CPG review process using CAPGO between 1999 and 2005, and data gathered in a separate PEBC survey during this time [25]. All respondents were clinicians involved in the care and treatment of patients with cancer.

### Outcome variables

Study outcomes were clinicians' perceptions of CPG quality, their endorsement of the CPGs, and their intentions to use the CPGs, and these were measured using the validated survey from the PEBC external review process, the CAPGO instrument, (see Table 1) [9]. Four domains of quality were assessed: rigour, acceptability, applicability, and comparative value. The rigour domain focused on clinicians' perceptions of the CPG rationale, quality of scientific methodology used to develop the CPG, and clarity of the recommendations. The acceptability domain targeted clinicians' perceptions of the acceptability and suitability of the recommendations, belief that they would yield more benefits than harms, and anticipated acceptance of recommendations by patients and colleagues. The applicability domain targeted clinicians' perceptions of the ease of implementing recommendations, considering the capacity to apply recommendations, technical requirements, organizational requirements, and costs. The comparative value domain asked clinicians for their perceptions of the recommendations relative to current standards of care. Clinicians' endorsement of the CPG (i.e., whether it should be approved) and their intentions to use the CPG in practice were assessed with single items. Quality, endorsement, and intentions scores ranged from one to five, with higher scores representing more favorable perceptions, higher endorsement, and greater intentions to use.

**Table 1:** The Clinicians' Assessments of Practice Guidelines in Oncology (CAPGO) survey

Item	Domain or Outcome
1. Are you responsible for the care of patients for whom this draft report is relevant? This may include the referral, diagnosis, treatment, or follow-up of patients. ('Yes', 'No' or 'Unsure'. If 'Yes', please answer the questions below.	NA

2. The rationale for developing a guideline, as stated in the 'Introduction' section of this draft report, is clear.	Quality

3. There is a need for a guideline on this topic.	Quality

4. The literature search is relevant and complete (*e.g.*, no key trials were missed nor any included that should not have been).	Quality

5. I agree with the methodology used to summarize the evidence.	Quality

6. The results of the trials described in this draft report are interpreted according to my understanding of the data.	Quality

7. The draft recommendations in this report are clear.	Quality

8. I agree with the draft recommendations as stated.	Acceptability

9. The draft recommendations are suitable for the patients for whom they are intended.	Acceptability

10. The draft recommendations are too rigid to apply to individual patients.	Applicability

11. When applied, the draft recommendations will produce more benefits for patients than harms.	Acceptability

12. The draft report presents options that will be acceptable to patients.	Acceptability

13. To apply the draft recommendations will require reorganization of services/care in my practice setting.	Applicability

14. To apply the draft recommendations will be technically challenging.	Applicability

15. The draft recommendations are too expensive to apply.	Applicability

16. The draft recommendations are likely to be supported by a majority of my colleagues.	Acceptability

17. If I follow the draft recommendations, the expected effects on patient outcomes will be obvious.	Acceptability

18. The draft recommendations reflect a more effective approach for improving patient outcomes than is current usual practice. (if they are the same as current practice, please tick NA).	Comparative value

19. When applied, the draft recommendations will result in better use of resources than current usual practice (if they are the same as current practice, please tick NA).	Comparative value

20. I would feel comfortable if my patients received the care recommended in the draft report.*	Endorsement

21. This draft report should be approved as a practice guideline.	Endorsement

22. If this draft report were to be approved as a practice guideline, how likely would you be to make use of it in your own practice?	Intentions to use in practice

23. If this draft report were to be approved as a practice guideline, how likely would you be to apply the recommendations to your patients?	Intentions to use with patients

*Items 1, 20, and 23 were not considered in this study.

### Predictor variables

This study analyzed two sets of predictor variables: clinician characteristics and clinician beliefs and attitudes. Clinician characteristics data, which included clinical discipline, gender, and average number of hours spent per week with research (as primary investigator, co-investigator in any cancer-related research study), were obtained from the PEBC database. Data on clinicians' beliefs about and attitudes towards CPGs were gathered in the Ontario physician survey [25]. This survey considered three belief domains: beliefs that CPGs are linked to change in practice, negative misconceptions regarding CPGs, and beliefs regarding CPGs as tools to advance quality. We also measured clinicians' overall attitudes towards CPGs (negative-positive). See Table 2.

**Table 2:** Six-year mean, year one mean, and annual change in quality, endorsement and intention scores

**Domain** **(Score Range)**	**Mean 6-Year Score** **(%)**	**Estimated Score Year 1** **(95% CI)**	**Annual Change** **(95% CI)**	**p**	**% Variance** **Clinicians**
Rigour (6–30)	26.2 (87.3)	25.7 (25.5, 30.0)	0.15 (0.10, 0.19)	<0.001	38.3

Acceptability (6–30)	23.6 (78.7)	23.0 (22.7, 23.3)	0.19 (0.13, 0.25)	<0.001	28.3

Applicability (4–20)	14.9 (74.5)	15.1 (14.8, 15.4)	-0.14 (-0.19, -0.09)	<0.001	27.8

Comparative Value (2–10)	6.8 (68.0)	6.6 (6.4, 6.8)	0.05 (0.01, 0.08)	0.009	23.8

Endorsement (1–5)	4.1 (82.0)	3.9 (3.9, 4.0)	0.02 (0.01, 0.04)	0.001	25.5

Intention to Use (1–5)	4.2 (84.0)	4.2 (4.1, 4.3)	-0.03 (-0.04, -0.01)	0.003	18.7

### Analyses

Most clinicians in the study rated more than one CPG, although the unit of analysis was the individual CPG. Consequently, the data set has a multilevel structure, and CPGs are nested within clinicians. Multilevel modeling was used to evaluate how CPG characteristics, clinical characteristics, clinical beliefs, and clinical attitudes predicted users' perceptions of CPGs over time, while appropriately accounting for the nested data structure [26]. Multilevel modeling quantifies similarity of ratings within clinicians and appropriately adjusts the statistical tests of the predictors. Specifically, a regression model for the effects of year and any additional predictors is estimated to describe the trends for the average clinician. These are known as the fixed effects. To accommodate variations among clinicians in their overall rating tendencies, each clinician is assumed to have his or her own intercept, reflected as a random deviation from the average intercept. The variance of these 'random effects' is estimated and, as a proportion of the total variance, reflects the percentage of variance accounted for after adjusting for the predictors. To facilitate interpretation of the intercept, analyses involving year were completed with the year centered on the first year of data (1999). Each predictor additional to year was tested in a separate analysis with year, the predictor, and the year × predictor interaction included. The interaction assesses whether the predictor affects change in ratings over time. Variations in the number of ratings per CPG are easily handled within the multilevel modeling framework.

## Results

### Sample

Between 1999 and 2005, 756 physicians participated in the evaluation of 84 specific cancer care CPGs developed in Ontario, yielding 4,091 CAPGO survey responses; more than 70% of clinicians rated more than one CPG. With respect to CPG characteristics, systemic therapy, radiation therapy, and surgery accounted for 58.3%, 15.5%, and 3.6% of the guidelines topics, respectively. The DSG representing the 'big four' cancer sites (breast, gastrointestinal, genitourinary, and lung) authored 54.8% of the CPGs.

With respect to clinician characteristics, medical oncologists, radiation oncologists, and surgeons accounted for 30.4%, 11.6%, and 38.6% of the participant sample, respectively, with other specialists accounting for the remaining 19.5% of the sample. Only 20.7% of the sample was women.

### Quality, endorsement, and intention to use in practice scores

Table 2 presents the mean ratings for each of the outcomes. The means for each of the measures were consistently high, and across the quality domains the six-year mean scores ranged from 68.0% to 87.3% of the total possible scores.

Table 2 also reports the estimated scores for each outcome variable for the first year (1999) and the annual changes with each subsequent year. With the exception of the applicability and intentions to use scores, there were small but statistically significant net gains in ratings, with the magnitude of change being between 0.02 (endorsement) and 0.19 (acceptability) per year. In contrast, small but statistically significant net losses were found for applicability ratings (-0.14) and intention to use ratings (-0.03) per year. The proportions of variance in outcomes associated with differences among practitioners are also reported in Table 2.

### Impact of predictors

Additional File 1 reports the main effects of each predictor variable and the interaction between time and predictors for each of the outcome variables.

### Clinician characteristics

#### Clinician discipline

A significant main effect of clinician discipline was found for the rigour (p = 0.01) and applicability (p < 0.038) scores. Rigour scores given by medical oncologists were highest, by radiation oncologists and surgeons were in the middle, and by 'other' specialists were lowest. Applicability scores were highest for medical oncologists and radiation oncologists compared to surgeons and 'other' specialists.

**Figure 1:**
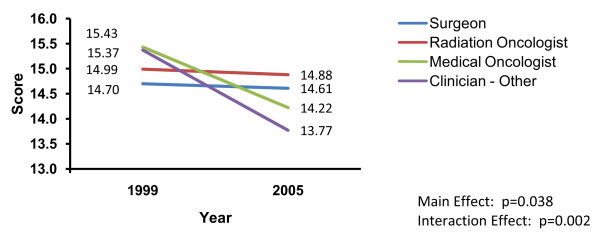
**Time by clinician discipline interaction on clinicians' ratings of CPG applicability**.

A significant time by clinician discipline interaction emerged for the applicability score (p = 0.002). Beginning in 1999, medical oncologists and 'other' clinicians had higher applicability scores in contrast to radiation oncologists and surgeons. However, this pattern reversed over time with medical oncologists and 'other' clinicians showing the largest decline in scores in contrast to radiation oncologists and surgeons, where virtually no change was seen (see Figure 1).

#### Research involvement

A significant time by research involvement interaction was found for the applicability (p < 0.006) and comparative value (p < 0.027) scores. With the comparative value rating, clinicians' initial scores in 1999 were virtually identical but, over time scores varied among the disciplines as a function of the amount of time devoted to research. Specifically, while little change was seen over time with those who devoted little or a moderate amount of time to research, a sharp decline in comparative value scores was seen in those who devoted a large amount of time.

In contrast, with the applicability score, in 1999 these ratings were higher for those who devoted a large amount of time to research compared to those who devoted less, with the inverse emerging by 2005.

#### Gender

There was significant main effect for gender (favouring females) (p = 0.034) and a significant time by gender interaction (p = 0.045) for intention to use CPGs. Females were more likely to report greater intention to use CPGs compared to males in 1999. However, this pattern reversed by 2005.

### Impact of clinician perceptions and attitudes

#### Belief CPGs linked to change

Comparative value scores diverged over time as a function of clinicians' belief that CPGs are linked to change. Specifically, comparative value scores in 1999 were lower for clinicians who believed CPGs were linked to change compared to those who believed practice could remain unchanged. A reverse pattern was found by 2005, with a larger difference found among the groups (p < 0.036).

#### Misconception beliefs about CPGs

**Figure 2:**
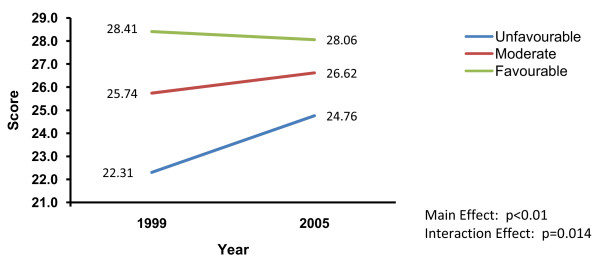
**Time by misconception beliefs about CPGs interaction on clinicians' ratings of CPG rigour**.

Significant main effects for CPG misconception beliefs and significant time by CPG misconception belief interactions emerged on rigour (p < 0.01 and p = 0.014, respectively), acceptability (p < 0.01 and p = 0.006, respectively), comparative value (p < 0.01 and p ≤ 0.006, respectively), CPG endorsement (p < 0.01 and p = 0.002, respectively), and intention to use CPGs (p < 0.01 and p = 0.003, respectively) scores. Very common patterns of main effects and interactions were found for these outcomes. Specifically, scores were higher among clinicians with more favourable beliefs (i.e., fewest misconceptions), followed by those with moderate beliefs, and lowest for those with more unfavourable beliefs (i.e., most misconceptions). However, in contrast to those clinicians with more favourable or moderate beliefs (where either no difference or only small changes in scores were observed over time), scores increased over time among clinicians who had less favourable beliefs about CPGs. Thus, differences in scores between groups became smaller over time due to increases in quality, endorsement, and intention scores for those holding the most unfavourable beliefs. Figure 2 illustrates this pattern, using the interaction findings related to clinicians' CPG rigour ratings as the exemplar.

#### Beliefs CPGs advance quality

Significant main effects were found for rigour (p < 0.01), applicability (p < 0.01), acceptability (p < 0.01), and intention to use scores (p < 0.01) on clinicians' belief that CPGs advance quality. In all cases, scores were higher among clinicians who were more likely to believe CPGs were good scientific tools to advance quality, followed by those with moderate beliefs, and lowest for those least likely to believe CPGs were good scientific tools to advance quality.

**Figure 3:**
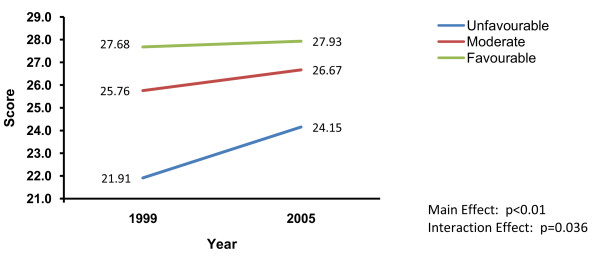
**Time by beliefs that CPGs advance quality interaction on clinicians' ratings of CPG rigour**.

Main effects were subsumed by significant time by beliefs interactions for the rigour (p < 0.036) and intention to use (p < 0.024) scores. The pattern of interaction was similar in both cases. Scores increased over time for clinicians who were least likely to perceive CPGs as good scientific tools to advance quality. In contrast, for clinicians with more favourable or neutral beliefs, rigour and intention to use scores remained stable or changed slightly. Thus, over time, the differences between groups became smaller, again due to increases in scores by those holding the most unfavourable beliefs. Figure 3 illustrates this pattern using the interaction findings of clinicians' CPG Rigour ratings as the exemplar.

#### Clinician attitudes about CPGs

Significant main effects were found with CPG attitude scores for rigour (p < 0.01), acceptability (p < 0.01), comparative value (p < 0.01), endorsement (p < 0.01), and intention to use CPGs (p < 0.01) scores. In all cases, scores were higher among clinicians who held more positive attitudes, followed by those who held neutral attitudes, and lowest for those who held more negative attitudes.

**Figure 4:**
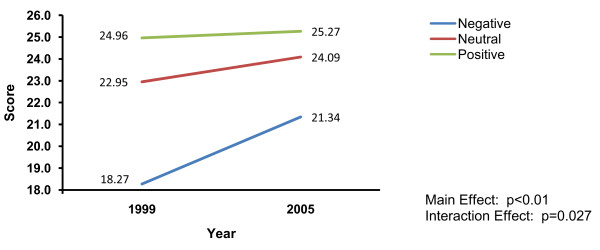
**Time by clinician CPG attitudes interaction on clinicians' ratings of CPG acceptability**.

Main effects were subsumed by significant time by clinician attitude interactions for the acceptability (p < 0.027), comparative value (p < 0.042), and endorsement ratings (p < 0.005). Again, patterns were extremely similar across the outcome measures. Among clinicians with very positive or moderately positive attitudes towards CPGs, there was little change in scores over time (scores remained very high). In contrast, increases in scores were observed over time among clinicians whose general attitudes were less positive. Thus, as has been seen elsewhere, the differences among groups lessened over time. Figure 4 illustrates this pattern using the interaction findings of clinicians' CPG acceptability ratings.

## Discussion

This study examined the influence of clinician characteristics, beliefs, and attitudes on clinicians' ratings of CPGs over time in a formal integrated healthcare system. PEBC cancer CPGs were evaluated as being of high quality. They were strongly endorsed, and clinicians reported high intention to use them in practice. Scores increased over time for rigour, acceptability, comparative value, and intention to use scores, whereas significant annual declines were found for endorsement and applicability scores. However, the absolute annual changes were small, possibly reflecting a ceiling effect due to the high ratings overall.

The range in variance accounted for by differences among practitioners was 23.8% to 38.3% for the quality domains, 25.5% in the endorsement item, and 18.7% in the intention to use in practice item. These values are similar to those found in previous studies [9], and suggest understanding the characteristics of clinician stakeholders are important to better understand and predict ratings of and intention to use recommendations.

The effects of the predictors were similar across outcome measures. The ratings of specific CPG's were higher among clinicians who held the more favourable beliefs, more positive attitudes, and had fewer negative misconceptions about CPG's. That is, general beliefs and attitudes appear to reflect a general orientation that strongly influences reactions to specific documents. However, we also found ratings of specific CPGs tended to improve over time for clinicians with the least favourable general beliefs and most negative attitudes. These data provide important lessons regarding the application of evidence into practice.

Specifically, the data identify factors that may be useful for interventions or system redesign aimed to promote evidence-informed decisions. For example, our study suggests that continued engagement of clinicians who are least receptive to cancer CPGs may be worthwhile. Perhaps with increased exposure to cancer CPGs through external review processes, the use and application of cancer CPGs in their clinical setting, CPGs as an educational intervention, and/or exposure to clinical policy, clinicians more wary of cancer CPGs become increasingly convinced of the role of these tools. It may also be that the influence of clinicians' negative preconceptions about CPGs is becoming less as evidence-based CPGs become increasingly established in the organizational and clinical culture of cancer care. Purposefully creating repeated opportunities for engagement among stakeholders in the cancer CPG enterprise, including the least supportive stakeholder group, may prove to be an effective component to an overall implementation strategy to facilitate the uptake of evidence. However, our unexpected findings of differences between the intentions of women and men to use CPGs over time, suggest further study is required to be able to adequately tailor interventions so that all stakeholders feel engaged.

These data also highlight the value of the methodology we used to examine, from a longitudinal perspective, the interface between knowledge products (i.e., the guideline) and the users of the knowledge (i.e., the clinicians). We found that ratings of CPG applicability and comparative value declined over time among clinicians who were more involved in research. Low scores on the applicability domain were not particularly surprising, as this has been found elsewhere. For example, in a review of 32 oncology guidelines, Burgers *et al*. found applicability scores to be extremely low, averaging 25.8% [27]. However, the decline over time was unexpected, and we can only speculate as to why this might be so. More recent cancer CPGs tend to have an increased focus on novel therapeutic agents and technologies, for which there is often an incomplete evidentiary basis or uncertainty regarding issues of implementation and public policy. Thus, this may place into question the value and role of these treatment options.

The dramatic shift in DSG portfolios towards CPGs for novel therapies may also explain the finding that ratings of CPG applicability were more likely to decline over time among medical oncologists than other specialties. Medical oncologists are primarily responsible for the evaluation of novel chemotherapy agents. From a clinical practice perspective, physicians want to advocate for their patients, and CPGs can provide an avenue to enable the evidence to support this goal. However, tension is provoked in the Ontario cancer care system, a publicly funded system, because the CPGs are also formally used by government in decisions about which drugs should be paid and made accessible to patients. Here, failure to get access to promising but not proven care options due to budget constraints or failure to meet evidentiary thresholds can render the CPG irrelevant. These findings highlight the importance of understanding CPGs in a larger healthcare context, changes to the context, and the conflicts that sometimes result.

There are limitations to this work. The findings of this study are constrained to individuals who participate, in some fashion, in the CPG enterprise. We have little data on those who have chosen never to exercise that opportunity. It is not possible, therefore, to predict the beliefs, intentions, and characteristics of the non-responders. It may be useful to explore failure to participate to better understand if it is driven by a lack of support for an evidence-based framework to support decision making or other non-related features (*e.g.*, limited time). A separate project, in progress, is exploring these issues and in particular links between intensity of participation and patterns of CPG quality and intentions to use CPGs.

A second limitation is that the analysis stopped at clinicians' intentions to use CPGs rather than evaluate actual use (*e.g.*, prescription patterns for chemotherapy, radiotherapy regimens as notes in patient file). Previous research has demonstrated reasonably moderate correlations between intention measures and behavioral measures in the healthcare literature, albeit with some significant methodological caveats [28]. Nonetheless, this work gives us some reassurance about the applicability of our findings to contribute the larger evidence utilization and application research literature. Regardless, clinical decisions and clinical outcomes are the desired and gold standard for evaluation; our objectives are to complete that task in the next steps of this program of research by focusing on how these evaluations are related to treatment decisions related to CPGs.

## Conclusion

We have successfully examined the temporal trends in clinicians' evaluations of CPGs as well as clinician characteristics that might impact these changes. This study highlights the importance of construing quality in terms of clinicians' perceptions, rather than only the objective properties of guidelines. The results support the view that the quality and effectiveness of CPGs are best understood in terms of the contexts where they are used and the characteristics, beliefs, and attitudes of the users.

## Competing interests

The authors declare that they have no competing interests.

## Authors' contributions

MB and SH conceived and designed the project, oversaw the analysis and interpretation of the data, drafted and revised the manuscript, and have given final approval of the submitted manuscript. MA-M and JY contributed to the design of the project, analyzed the data, and contributed to the writing and revision of the manuscript, and have given final approval of the submitted manuscript. MB acquired the data. This project contributed to the Master's degree educational requirements of Mona Abdel-Motagally and Jennifer Yee.

## Supplementary Material

Additional file 1 **Significant predictor main effects (top) and significant predictor by time interactions (bottom) for outcome measures**. This table provides the results of the statistical analyses testing the main effects of each predictor variable and the interactions between the predictor variable by time for each of the outcome measures. Click here for file
