# Supplementary material for: Clinicians' evaluations of, endorsements of, and intentions to use practice guidelines change over time: a retrospective analysis from an organized guideline program
Source: Implement Sci. 2009 Jun 28;4:34. doi: 10.1186/1748-5908-4-34 (PMC2715368; doi:10.1186/1748-5908-4-34)
Supplement: Additional file 1 — Significant predictor main effects (top) and significant predictor by time interactions (bottom) for outcome measures. This table provides the results of the statistical analyses testing the main effects of each predictor variable and the interactions between the predictor variable by time for each of the outcome measures. [file 1748-5908-4-34-S1.doc]

**Additional File 1:** **Significant predictor main effects (top) and significant predictor by time interactions (bottom) for outcome measures**

| **Predictor**  **Variables** | **Predictor Variable**  **Categories or Ranges/Means** | **Outcome Variables^1^** | | | | | | |
| --- | --- | --- | --- | --- | --- | --- | --- | --- |
|  |  | Rigour | | Acceptability | Applicability | Comparative  Value | Endorsement | Intentions  To Use |
| **Clinician Characteristics** | | |  | | | | | |
| Discipline | Medical Oncology, Radiation Oncology, Surgery, Other | p<.01  p=.058 | | ns  ns | p=.038  p=.002 | ns  ns | ns  ns | ns  ns |
| Research Involvement | Min: 0 Max: 70 Mean: 6  less involvement – more involvement | ns  ns | | ns  ns | ns  p=.016 | ns  p=.027 | ns  ns | ns  ns |
| Gender | Male, Female | ns  ns | | ns  ns | ns  ns | ns  ns | ns  ns | p=.034  p=.045 |
| **Clinician Beliefs (Unfavourable-Favourable) and Attitudes (Negative-Positive) about CPGs^2^** | | | | | | | | |
| CPGs Linked to Change | Min: 2 Max: 10 Mean: 7  unfavourable beliefs – favourable beliefs | ns  ns | | ns  ns | ns  p=.036 | ns  ns | ns  ns | ns  ns |
| CPG Misconceptions | Min: 9 Max: 25 Mean: 18  unfavourable beliefs – favourable beliefs | p<.01  p=.014 | | p<.01  p=.006 | ns  ns | p<.01  p=.006 | p<.01  p=.002 | p<.01  p=.003 |
| CPGs Advance Quality | Min: 6 Max: 15 Mean: 12  unfavourable belief – favourable beliefs | p<.01  p=.036 | | p<.01  ns | p<.01  ns | ns  ns | ns  ns | p<.01  p=.024 |
| General Attitudes about CPGs | Min: 5 Max: 15 Mean: 12  negative attitudes – positive attitudes | p<.01  ns | | p<.01  p=.027 | ns  ns | p<.01  p=.042 | p<.01  p=.002 | p<.01  ns |

^1^. Data from CAPGO Survey. ^2^ Data from Ontario Physicians Survey. ns = not significant . min=minimum score. max=maximum score.
